# On the origin of euphyllophyte roots: hypotheses from an Early Devonian *Psilophyton*

**DOI:** 10.1093/aob/mcaf121

**Published:** 2025-06-13

**Authors:** Jeffrey B Doran, Alexandru M F Tomescu

**Affiliations:** Alplaus, NY 12008, USA; Department of Biological Sciences, California State Polytechnic University—Humboldt, Arcata, CA 95521, USA

**Keywords:** Fossil, *Psilophyton*, euphyllophyte, rooting structures, root evolution, gravity-induced morphology, Devonian, spinescent emergence

## Abstract

**Background and Aims:**

Rooting structures have been documented in different Early Devonian plants, including rhyniophytes and zosterophylls. However, the basal euphyllophytes, the plexus from which modern ferns, equisetophytes and seed plants evolved, are the only group with no known rooting structures in Early Devonian representatives. We revisit the early euphyllophyte *Psilophyton crenulatum*, whose emergences have implications for rooting structure evolution within the clade.

**Methods:**

Well preserved Early Devonian (earliest Emsian) material from the Val d’Amour Formation in New Brunswick (Canada) was freed from the rock matrix using acid. Over 2000 emergences from 28 randomly selected *P. crenulatum* axes were characterized qualitatively and quantitatively in terms of morphology and distribution.

**Key Results:**

The features of the emergences are more consistent with a rooting function than with any other possible role: irregular morphology, including forms with complex branching; anatomy involving external layers of thin-walled cells; apical meristematic growth that allowed for branching; terminations in filiform rhizoid-sized tips; and vertically polarized distribution, with nearly twice as many emergences on the lower sides of axes compared with the upper sides. The absence of any other potential rooting structures associated with *P. crenulatum* also supports a rooting function for the emergences.

**Conclusions:**

If the emergences of *P. crenulatum* had a rooting function, they are the oldest euphyllophyte rooting structures. They are also a novel, *sui generis* type of such structures among Early Devonian plants. They also provide the oldest direct evidence for gravity-induced morphological features in euphyllophytes. If they evolved from emergences like those of *P. crenulatum*, then euphyllophyte roots probably inherited from them apical growth and branching, and gene networks responsible for production of tip-growing filamentous cells. Progressive increase in size of such emergences could have increased auxin fluxes, leading to specification of vascular connections to subtending axes.

## INTRODUCTION

Rhizoid-bearing projections, dome-shaped or ridge-shaped, produced on rhizomatous axes are the simplest rooting structures documented in polysporangiophytic early land plants. Such rhizoid-bearing projections are known in the protracheophytes and rhyniopsids preserved in the Lower Devonian (Pragian) Rhynie chert (Kidston and Lang, 1917, 1920*a*; Edwards, 1986; Remy and Hass, 1996; Kerp *et al*., 2001; Kenrick and Strullu-Derrien, 2014; Kerp, 2017; Hetherington and Dolan, 2017*a*). More complex rooting structures documented in early vascular plants consist of vascularized branches that are produced in most cases by K-branching or from subaxillary meristems and can have positively gravitropic growth. Such branches have been reported in zosterophylls and lycopsids that span the Lower to early Middle Devonian (Lochkovian to Eifelian) (e.g. Edwards *et al*., 1989; Gensel *et al*., 2001; Matsunaga *et al*., 2017; see also Table 1). The oldest roots are known in Early Devonian drepanophycalean lycopsids dated to the Lochkovian–Pragian boundary (Matsunaga and Tomescu, 2016, 2017, Noetinger *et al*., 2021). Here and throughout, rooting structure is a general term that refers to any structure involved in anchorage and water absorption, including roots as well as structures that may be more or less root-like in their morphology. Root or true root refers to axial organs that lack appendicular organs (leaves) and possess positive gravitropic response, a root cap and endogenous origination from subtending organs (Gensel *et al*., 2001; Raven and Edwards, 2001; Kenrick and Strullu-Derrien, 2014; Matsunaga and Tomescu, 2016; Hetherington and Dolan, 2017*b*, 2018); additionally, all roots share steles with exarch maturation of the primary xylem.

**Table 1.** Rooting structures of early polysporangiophytes.

Species	Rhizoids	Rooting structures	Age	Taxonomy	References
**Protracheophytes and rhyniopsids**					
*Aglaophyton majus*	Unicellular, 30–50 µm diameter, borne on rooting structures	Multicellular unvascularized projections (at contact with substrate), sometimes conjoined along lower side of rhizomatous portions	Pragian	Protracheophyte	Edwards, 1986; Remy and Hass, 1996; Hetherington and Dolan, 2017*a*, *b*
*Horneophyton lignieri*	Unicellular, 50 µm diameter, borne on lower surface of rooting structures	Inflated, tuberous, sometimes lobed base of sporophyte	Pragian	Horneophytopsid	Kidston and Lang, 1920*a*; Crane *et al*., 2004; Hetherington and Dolan, 2017*a*, *b*
*Nothia aphylla*	Unicellular, 30–50 µm diameter, borne on rooting structures	Multicellular unvascularized longitudinal ridges on lower side of rhizomatous axes	Pragian	?	Gensel *et al*., 2001; Kerp, 2017
*Rhynia gwynne-vaughanii*	Unicellular, 20–30 µm diameter, borne on rhizomatous axes and their rooting structures	Multicellular unvascularized hemispherical projections protruding laterally from rhizomatous axes; projections of same type bear stomata on the upright axes	Pragian	Rhyniopsid	Kidston and Lang, 1917; Crane *et al*., 2004; Hetherington and Dolan, 2017*a*, *b*
*Stockmansella* (*Taeniocrada*) *langii*	Not documented	Positively gravitropic dichotomously branching axes (0.3–1.0 mm thick) diverging from rhizomatous portions	Late Pragian–Emsian	Rhyniopsid	Schweitzer, 1980; Fairon-Demaret, 1985, 1986; Crane *et al*., 2004
**Zosterophylls and allied taxa**					
*Anisophyton gothani*	Not documented	Branches from subaxillary tubercles, dichotomously branched, ∼2 mm thick	Emsian	Zosterophyll?	Remy *et al*., 1986a, b
*Anisophyton potoniei*	Not documented	Branches from subaxillary tubercles, dichotomously branched, ∼0.5–0.8 mm thick	Emsian	Zosterophyll?	Remy *et al*., 1986*a*, *b*
*Bathurstia denticulata*	Not documented	Positively gravitropic axes (1) produced by K-branching, <3 mm thick, and (2) diverging at right angle from rhizomatous axes, 1.0–2.3 mm thick	Pragian	Zosterophyll	Kotyk and Basinger, 2000; Gensel *et al*., 2001
*Crenaticaulis verruculosus*	Present?	(?Positively gravitropic) axes (1) developed from subaxillary tubercles, 0.4–1.0 mm thick and (2) not associated with branching, dichotomously branched, 0.5 mm thick	Late Emsian	Zosterophyll	Banks and Davis, 1969; Gensel *et al*., 2001; Crane *et al*., 2004
*Deheubarthia splendens*	Not documented	(?Positively gravitropic) axes developed from subaxillary tubercles, 2 mm thick	Late Lochkovian–Pragian	Zosterophyll	Edwards *et al*., 1989
*Gosslingia breconensis*	Not documented	(?Positively gravitropic) axes developed from subaxillary tubercles, 0.4–1.4 mm thick	Pragian	Zosterophyll	Edwards, 1970; Edwards *et al*., 1989
*Hsua robusta*	Not documented	Dichotomous root-like branches 0.3–1.0 mm thick (<2.5 mm at base) borne on axes, some produced by K-branching	Late Pragian–early Emsian	Zosterophyll?	Li 1992
*Margophyton goldschmidtii*	Not documented	Subaxillary tubercles	Pragian–Emsian	Zosterophyll	Kenrick and Edwards, 1988
*Sawdonia ornata*	Not documented	(?Positively gravitropic) axes (1 mm thick) produced by K-branching	Early–mid Emsian	Zosterophyll	Rayner, 1983
*Tarella trowenii*	Not documented	(?Positively gravitropic) axes (0.5 mm thick) produced by K-branching	Pragian	Zosterophyll	Edwards and Kenrick, 1986
*Thrinkophyton formosum*	Not documented	Subaxillary tubercles	Late Lochkovian–Pragian	Zosterophyll	Kenrick and Edwards, 1988
*Trichopherophyton teuchansii*	Unicellular 30–40 µm diameter, borne on putatively rhizomatous axes	?	Pragian	Zosterophyll	Niklas and Banks, 1990; Lyon and Edwards, 1991; Edwards, 2004
*Zosterophyllum myretonianum*	Not documented	Positively gravitropic axes (1–3 mm thick) produced by K-branching of rhizomatous portions	Lochkovian	Zosterophyll	Walton, 1964
*Zosterophyllum shengfengense*	Not documented	Positively gravitropic axes (0.3–0.7 mm thick) produced by K-branching of rhizomatous portions	Lochkovian	Zosterophyll	Hao *et al*., 2010; Hao and Xue, 2013
*Zosterophyllum* sp. nov.	Not documented	Positively gravitropic axes 1 mm thick diverging from rhizomatous portions (possibly by K-branching)	Pragian	Zosterophyll	Gensel *et al*., 2001
New Beartooth Butte Formation plant	Not documented	(? Positively gravitropic) axes (5 mm thick) produced by K-branching	Late Lochkovian–early Pragian	zosterophyll	S.R. El-Abdallah Cal Poly Humboldt, USA, unpublished
**Lycopsids**					
*Asteroxylon mackiei*	Absent	(1) Positively gravitropic stems (root-bearing axes, 3.5 mm diameter) with reduced or absent leaves that produce (2) weakly gravitropic axes <2 mm diameter by anisotomous dichotomy	Pragian	Lycopsid	Kidston and Lang, 1920*b*, 1921; Hetherington *et al*., 2021
*Drepanophycus qujingensis*	Not documented	Stem-borne roots, < 3 mm thick, branched dichotomously	Pragian toearliest Emsian	Lycopsid	Li and Edwards, 1995; Gensel *et al*., 2001; Xue *et al*., 2016
*Drepanophycus spinaeformis*	Not documented	Positively gravitropic leafless axes (1) produced by K-branching of stems, 1.5–2.5 mm thick, and (2) diverging at right angle from stems, 1.2–1.8 mm thick	Pragian	Lycopsid	Rayner, 1984; Gensel *et al*., 2001
*Sengelia* (*Drepanophycus*) *devonica*	Not documented	(1) (? Positively gravitropic) leafless axes produced by K-branching of stems, 4–11 mm thick, bearing (2) non-gravitropic dichotomously branching roots, 0.5–1.0 mm thick	Late Eifelian	Lycopsid	Schweitzer and Giesen, 1980; Gensel *et al*., 2001; Hao and Xue, 2013; Matsunaga and Tomescu, 2017
*Sengelia radicans*	Absent	(1) Positively gravitropic leafless axes produced by K-branching of stems, 2–8 mm thick, bearing (2) non-gravitropic dichotomously branching roots, 0.4–0.7 mm thick	Late Lochkovian–early Pragian	Lycopsid	Matsunaga and Tomescu, 2016, 2017

Question marks imply some level of uncertainty in the interpretation of structures.

Compared with the numerous records of rooting structures in zosterophylls, early lycopsids and other early tracheophytes, the euphyllophyte clade presents an intriguing situation. Despite a growing number of species reported from Lower Devonian deposits, there is a striking lack of good evidence for euphyllophyte rooting structures until the Middle Devonian (Eifelian–Givetian) (Fairon-Demaret and Li, 1993; Giesen and Berry, 2013; Hetherington *et al*., 2020) and for unequivocal roots until the Late Devonian (Frasnian, Xu *et al*., 2017). A member of the large genus *Psilophyton* now provides data that may fill the gap in the early fossil record of euphyllophyte rooting structures.

The genus *Psilophyton* groups at least 14 species ranging in age from Lower to early Middle Devonian (Pragian to early Eifelian) that include some of the oldest known euphyllophytes, central to our understanding of the early evolution of the clade. Diverse species of *Psilophyton* have contributed data on the vegetative anatomy of early euphyllophytes (Banks *et al*., 1975; Gensel, 1979; Trant and Gensel, 1985), their sporangium structure (Banks *et al*., 1975; Noetinger *et al*., 2018), spore wall structure and development (Banks *et al*., 1975; Doran, 1980; Gensel and White, 1983; Balme, 1995; Gerrienne, 1995; Noetinger *et al*., 2018) and the early evolution of water conduction (Cook and Friedman, 1998; Wilson, 2016), wound responses (Banks, 1981; Banks and Colthart, 1993) and anti-herbivore defences (Colston *et al*., 2023).

*Psilophyton crenulatum* is the species best characterized morphologically in this genus. Described a while ago by Doran (1980), *P. crenulatum* is also, along with *P. primitivum*, the oldest species in the genus. *Psilophyton crenulatum* is notable for the wealth of information it has provided on the morphology of early euphyllophytes. Its highly variable branching patterns demonstrate developmental plasticity and suggest considerable potential as the starting point for the evolution of different branching architectures.

Like many early tracheophytes, *P. crenulatum* bears spinescent emergences on its axes (Doran, 1980). In different species and genera, such emergences have been hypothesized or demonstrated to have filled one or more of several possible functions: increasing photosynthetic capacity; shading of photosynthetic tissues; decreasing transpiration; herbivore deterrence; assistance in plant support; and excretion (Kevan *et al*., 1975; Edwards, 1993; Edwards and Selden, 1993; Hanley *et al*., 2007; Colston *et al*., 2023). The emergences of *P. crenulatum*, numerous and relatively large, have not elicited comments on function up until now. Here, we revisit *P. crenulatum*, characterizing in detail the distribution, morphology and anatomy of its emergences. Alongside reappraising the geological context and taphonomy of *P. crenulatum*, we provide evidence that supports interpretation of its emergences as rooting structures, and we discuss this hypothesis in the context of the evolution of tracheophyte rooting structures.

## MATERIALS AND METHODS

### Geological setting

**Fig. 1.**
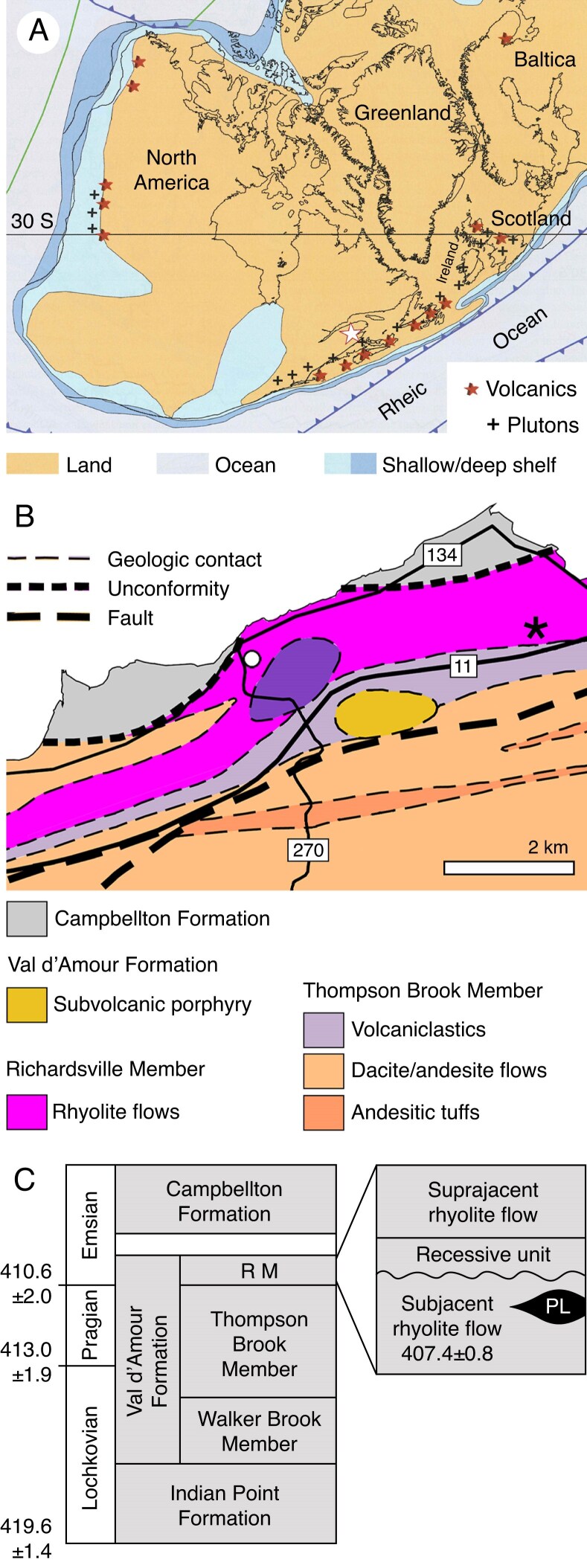
Place of the Atholville locality in space and time. (A) Palaeogeographic reconstruction of western and central Laurussia in the Early Devonian (Emsian, 400 Ma) (modified from Torsvik and Cocks, 2016, with permission). The white star indicates the approximate location of Atholville (New Brunswick, Canada). Note the proximity of Atholville to evidence of volcanism and its position relative to Scotland, where the Rhynie and Windyfield cherts host early land plants with rooting structures preserved in hydrothermally influenced environments. (B) Geological context of the Atholville locality where *Psilophyton crenulatum* lenses (white circle) are located in the rhyolite flows of the Richardsville Member of the Val d’Amour Formation. The asterisk marks the location of samples that provide the radiometric age of rhyolites and the numbers in rectangles are road numbers. Modified from Wilson *et al*. (2005) with permission. (C) Summary stratigraphy of Early Devonian units in the Atholville–Campbellton–Dalhousie area. PL indicates the position of the *P. crenulatum* lenses in the Richardsville member. Modified from Kennedy *et al*. (2013) with permission. Absolute ages are based on Cohen *et al*., 2013 (updated 2024).

The Late Silurian–Early Devonian interval witnessed the closing of the Rheic Ocean due to the convergence of the eastern edge of Laurentia and the western edge of Avalonia (Fig. 1A). As the two plates collided, marking the initiation of the Acadian Orogeny, subduction-associated volcanic activity produced the sequence of volcanic rocks and volcaniclastic deposits exposed in New Brunswick and the surrounding region of eastern Canada (Wilson *et al*., 2004, 2005; Kennedy and Gibling, 2011; Torsvik and Cocks, 2016). When *Psilophyton crenulatum* was first described from strata of this sequence (Doran, 1980), few publications had addressed the geology of the layers that hosted the fossils. The publications were broad surveys that grouped the Silurian and Devonian volcanic and volcaniclastic rocks of eastern Canada into a single unit called the Dalhousie Group (Logan *et al*., 1863; Hind, 1865; Clark, 1909; Howard, 1926; Alcock, 1935; Dineley and Williams, 1968; Greiner, 1970). Wilson *et al*. (2004, 2005) later introduced the Val d’Amour Formation for these strata in New Brunswick (Canada).

The Val d’Amour Formation encompasses ∼5800 m of Devonian volcanic rocks in the Atholville–Campbellton–Dalhousie region (New Brunswick, Canada; Fig. 1B). The oldest beds of the formation comprise the Walker Brook Member, which conformably overlies the Silurian–Devonian Indian Point Formation (Fig. 1C). The environments in which rocks of the Walker Brook Member accumulated transition from marine in the east (implying that during the Lochkovian the Rheic Ocean made its way into a coastal cove perhaps akin to present-day Chaleur Bay) to terrestrial headlands in the west (Wilson *et al*., 2005). Overlying the Walker Brook Member is the Thompson Brook Member, consisting of subaerial lava flows, tuffs and volcanoclastics (Fig. 1B, C). This member also includes the world’s oldest coaly shales accumulated during the Pragian (Kennedy *et al*., 2013), before deposition of the uppermost, Richardsville, Member of the Val d’Amour Formation.

**Fig. 2.**
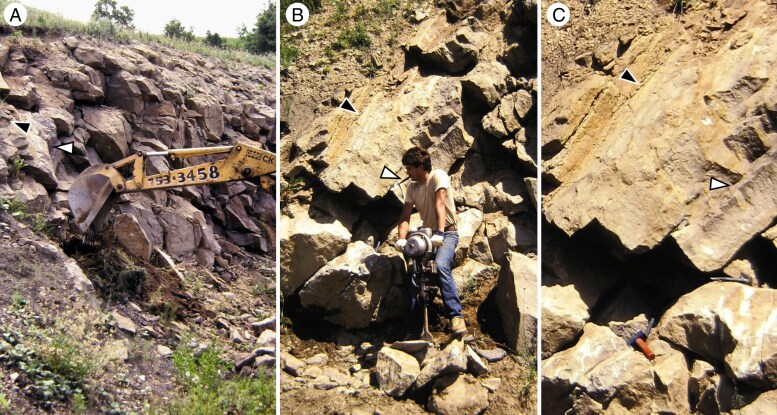
The Atholville outcrop of the Richardsville Member in 1977 and position of the *Psilophyton crenulatum* lens. (A) General view with backhoe bucket. (B, C) Details of (A) with the *P. crenulatum* lens (white arrowheads) in the subjacent rhyolite flow with respect to the base of the recessive sequence (black arrowheads; refer also to Fig. 1C). The sledgehammer in (C) is 30 cm long.

The Richardsville Member consists of ∼920 m of predominantly flow-layered rhyolite (Fig. 1B). Near Sugarloaf Mountain, just southeast from the *P. crenulatum* outcrop, some of the rhyolites exhibit mottled alteration suggesting local hydrothermal activity (Wilson *et al*., 2005). Towards the upper third of the Richardsville Member, lithology demonstrates a short period of erosion followed by deposition of a tuffaceous recessive sequence that includes mudstones rich in plant fossils (Gensel *et al*., 1997, 2005) (Figs 1C and 2A, B). This recessive sequence is bounded by rhyolite flows, of which the subjacent one contains the *P. crenulatum* lenses, and the suprajacent adds 100+ metres of rhyolite at the top of the Richardsville Member (Figs 1C and 2A). This upper rhyolite flow is unconformably overlain by the Campbellton Formation, signalling the full manifestation of the Acadian Orogenic front in this area.

The *P*. *crenulatum* lenses lie unquestionably in the Richardsville Member of the Val d’Amour Formation (Wilson *et al*., 2005; R. A. Wilson, Geological Surveys Branch, New Brunswick Department of Natural Resources, New Brunswick, Canada, pers. comm.) (Figs 1C and 2A–C). These lenses are embedded in and bounded by rhyolites that underlie the plant-fossiliferous recessive sequence reported by Gensel *et al*. (1997, 2005) (Figs 1C and 2A). The *P. crenulatum* lenses are separated from the conglomerate that marks the base of the recessive sequence by about ∼1 m of rhyolite.

### Age and phytostratigraphic context of the *Psilophyton crenulatum* layers

Doran (1980) assigned an early to middle Emsian age to the *P*. *crenulatum* lenses and since then Wilson *et al*. (2005) dated the Richardsville rhyolites at 407.4 ± 0.8 Ma using U-Pb on zircon crystals (Fig. 1B, C). The zircon dating, along with the most recent and updated absolute ages of Devonian stages (Cohen *et al*., 2013, updated 2024), suggests that the most conservative age estimate of the *P. crenulatum* lenses is no younger than the earliest Emsian, very near the Pragian–Emsian boundary.

Wilson *et al*. (2005) identified >50 species of spores from the Val d’Amour Formation, suggesting a relatively diverse Pragian–early Emsian local flora, even though a complete account of all the plant megafossils is yet to be produced. The only plant-bearing strata older than the *P. crenulatum* lenses in this region are ∼1 km to the southeast, in the Pragian Thompson Brook Member of the Val d’Amour Formation, where coaly shales have yielded zosterophylls including *Sawdonia*-like axes, axes consistent with the form genus *Taeniocrada*, a *Spongiophyton*-like thallus, and a possible trimerophyte reported by Kennedy *et al*. (2013). These authors interpreted these Pragian strata as terrestrial sediments that became vegetated along the fringes of ponds, between individual flow events.

### Sample processing

The *P. crenulatum* axes were extracted from the rock matrix by bulk maceration of hand specimens ∼ 30 × 15 cm in size, using ∼50 % hydrofluoric acid. Once the matrix was completely dissolved, the remaining plant material was neutralized by several water rinses. The plant fossils were examined in water under a Leica MZ8 dissecting microscope. They formed mats of axes, which were disassembled starting from the top, one axis after another until all the axes were removed. The mats were disentangled under water using dissecting needles; one needle was used to lift an overlying axis while hooking the other dissecting needle on the base of a spine and gently pulling to see where the contiguous portions of the axis being lifted trailed through the remaining mat of axes. Because very few axes were totally free (untangled) to float out of the mat and remove, and because of the numerous spiny emergences that led axes to become snagged against each other, the separation of individual axes was a time-consuming task. Once an axis was disentangled it was carefully floated away from the rest of the mat and allowed to settle on a submerged flexible piece of screening, for support. The piece of screening was ultimately removed from the water bath with the plant axis settled on it.

Samples recovered in this way were allowed to air-dry before being re-examined under the dissecting microscope on both sides and photographed with either a Canon EOS 80D attached to the Leica MZ8, a Canon Power Shot SX260 digital camera or an iPhone XR positioned atop an eyepiece of the Leica MZ8. Samples were embedded in clear synthetic resin and some were photographed with a combination of reflected light and light that was bounced off a mirror to create an underlit glow around portions of the sample. The eyepiece graticule was a Leica P/N 10376119, a 12-mm graticule with 120 divisions. Graticule division width was calibrated using a Westcott Stainless Steel (no. H590-18) ruler. For scanning electron microscopy, specimens were air-dried, placed on a stub, sputter-coated with gold and studied using a Cambridge S-150 SEM. Some specimens illustrated here come from the samples of Doran (1980), which were processed using the methods noted there. Specimens will be deposited in the University of Kansas Biodiversity Institute collections.

### Observations, terminology and measurements

We examined a total of 2090 emergences on 28 randomly selected axes. For each emergence we recorded the following:

1. Whether the emergence was simple (unbranched) or complex (branched).
2. Completeness of the emergence. The incomplete simple and complex emergences were only used in determining general trends (e.g. in emergence density, distribution on upper or lower axis surfaces) or if contributing to noteworthy data. It should be mentioned that an incomplete simple emergence was one that had any portion of its length missing, even if this meant only the apical tens of microns. Thus, only emergences that were intact to their very tips were included in the count of complete simple emergences. Complex emergences were treated slightly differently since a complex emergence has multiple tips; complex emergences that had at least one of the multiple tips complete were counted as complete.
3. Location on axis: upper or lower surface of the axis (see below).
4. Orientation of the emergence relative to its subtending axis: pointing towards the tip, towards the base or straight out laterally; branching points along the axes helped establish which end was proximal and which was distal.
5. Whether the emergence showed any curvature.
6. Width of the emergence at base.
7. Width of the emergence at a distance of 1 mm from the base.
8. Thickness of the emergence tip (if complete).
9. Total length of the (complete) emergence.
10. Length of the emergence from the point where it measured 0.33 mm in width to its end; the purpose of this measurement was to provide a measure of their taper, specifically by seeing how long the filiform apical portion of the emergence was.
11. For branched emergences, the length between the base and the first branching point, and between successive branching points.

For the quantitative morphological features of the emergences, the statistical significance of differences (or lack thereof) was assessed based on comparisons of both data means (with the Aspin–Welch unequal-variance *t*-test) and distributions (with the Kolmogorov–Smirnov test).

## RESULTS

### Fossiliferous rock matrix and preservation of the *Psilophyton crenulatum* axes

#### Fossiliferous lenses: lithology and relationships with adjacent strata

The felsic rhyolite hosting the Psilophyton lenses (Fig. 2) is buff colour, fine- to medium-grained, very hard and difficult to split, even with pneumatic jackhammers (Fig. 2B, C). A few larger blocks of the rhyolite including fossiliferous lenses were dislodged using a backhoe (Fig. 2A), but not before a backhoe tooth was snapped off by the hard rhyolite. In contrast, the overlying layers of mudstones of the recessive sequence are well stratified, weathered, and yield easily to shovel, hammer and chisel.

**Fig. 3.**
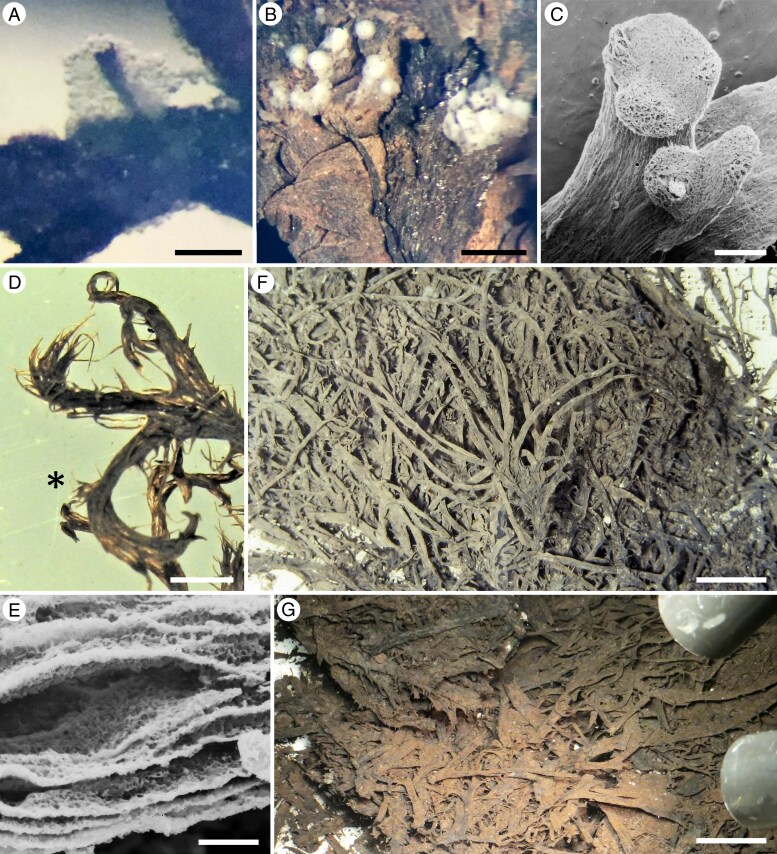
*Psilophyton crenulatum* sample processing and taphonomy. (A) Microcrystalline silica visible around axis after incomplete maceration. Scale bar = 1 mm. (B) Small detrital silica (round) and feldspar (rhomboidal) clasts (both white) visible after incomplete maceration. Scale bar = 1.5 mm. (C) Meristematic areas showing incipient branching at the tips of two sister axes, one (left, larger) with three branch primordia, and one (right, smaller) with two branch primordia. Note good preservation of individual cells. Scale bar = 150 µm. (D) Dense, delicate hair-like emergences in the apical region of upward-turned central branch and on small branch primordium on the lower branch (asterisk). Scale bar = 2 mm. (E) Cell walls of *P. crenulatum* sporangium cells with pock-marked surfaces possibly due to diagenesis. Scale bar = 20 µm. (F) and (G) Mats of compressed intertwined axes released by maceration from the siliceous rock matrix. Scale bars = 15 mm.

The lenses containing *Psilophyton* (Fig. 2) are grey to lightly rust-coloured, very fine-grained, unstratified and consist of secondarily silicified tuffaceous material formed from explosive volcanic glass particles, with small detrital clasts of clays and feldspars also present. Incomplete maceration of the fossiliferous matrix reveals some of these small detrital clasts (Fig. 3B), while remains of the secondary silica can be seen as a microcrystalline translucent coating surrounding the axes (Fig. 3A). The petrographic analysis did not reveal any discernible ‘soil’ (weathering surface) or distinct boundary layer between the fossiliferous lenses and the surrounding rhyolite.

#### Mode and quality of preservation of the *Psilophyton crenulatum* material

The plant material is preserved primarily as coalified compressions; the plants were covered by volcanic ash and then compressed. The robustness of the fossil axes and presence of fine details demonstrate a quality of preservation superior to that of typical compression fossils. The absence of any significant amounts of organic detritus in the rock matrix around the plant axes is consistent with minimal microbial decay prior to fossilization. Thus, although the material consists mostly of flattened axes, three-dimensionally preserved structures are also present, illustrating axis vasculature, internal anatomy of the emergences, apical cells of smaller lateral branches (Fig. 3C), young and mature sporangia, sporangial tapetum structure (many of them illustrated in Doran, 1980) and delicate hair-like emergences (Fig. 3D). The microcrystalline silica provided a moulding material favourable to preservation of the fine details but is also probably responsible for the minutely pockmarked aspect of many surfaces (Fig. 3E).

**Fig. 4.**
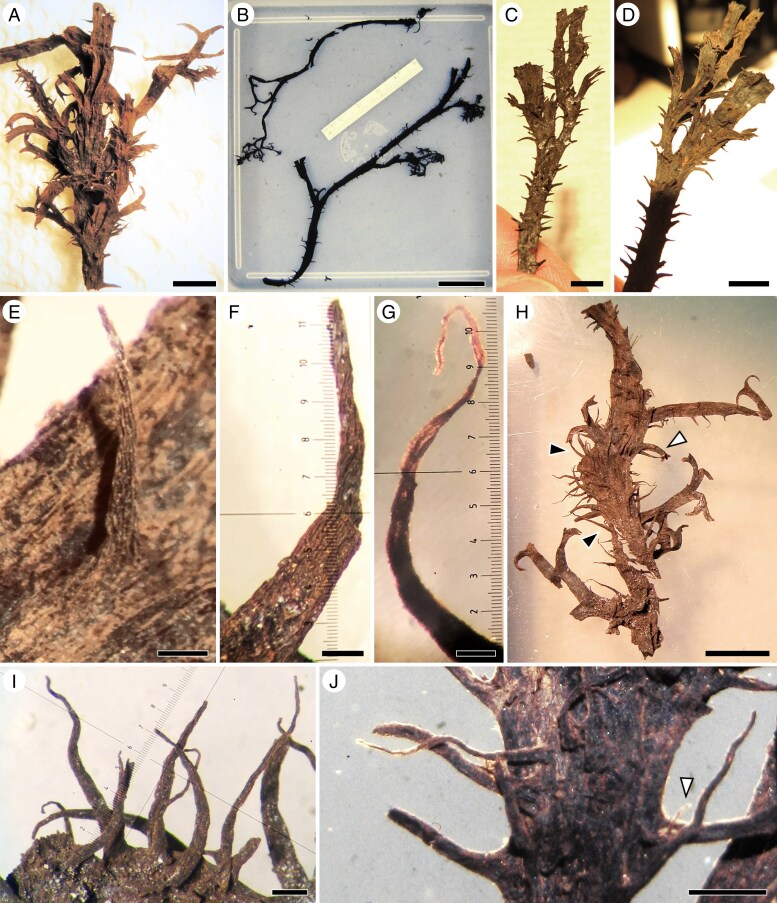
*Psilophyton crenulatum* simple spinescent emergences. (A) Profusely branched axis segment showing lower side (which faced the substrate in growth position) densely covered in simple and complex emergences. Branch tip with numerous emergences at top left are detailed in Fig. 9A. Scale bar = 5 mm. (B) Two large specimens (not considered in emergence counts and measurements): a vegetative branch truss (top) and a fertile one (bottom). Scale bar = 20 mm. (C, D) Upper side (which faced away from the substrate in growth position) (C) and lower side (D) of the same specimen. Note detrital clasts on the upper side [white specks in (C)] and amber-coloured cuticular material [on the two branches at top right in (D)] that helped in interpreting its orientation with respect to the substrate. Scale bars = 5 mm. (E) General brownish coloration typical of lower sides of most axes and amber-coloured cuticular remains also typically found on lower sides. Note sinistrally twisted simple emergence. Scale bar = 500 µm. (F) Sinistral twisting along the top half of a simple emergence. Note that the cells in the basal portion are aligned parallel with the edges of the emergence and the twisting begins somewhere around the emergence’s midpoint. Bimodal cell size suggesting diffuse elongation growth of the emergences is illustrated by the size contrast between the elongated cell (220 µm) with sigmoid shape present between the 7 and 8 markers of the graticule and the smaller oval-shaped cell (80 µm) at the 9 marker of the graticule. Scale bar = 300 µm. (G) Long (2.5 mm) narrow and sinuous simple emergence on an axis with long thin tip (22 µm diameter). Scale bar = 250 µm. (H) Branched axis segment illustrating emergence density and sinuousness on its lower side (between black arrowheads) immediately basal to, and continuing into, an area of profuse branching. Note small incipient branch (white arrowhead) ∼500 µm in diameter at the base, close in size to some of the stouter emergences observed on *P. crenulatum*. Scale bar = 5 mm. (I) Detail of the upper side of the axis in (H) showing numerous narrow and sinuous simple and complex emergences with long fine tips ∼22–27 µm in diameter. Scale bar = 500 µm. (J) Detail of axis with several sinuous, slender simple emergences. Note small and exceedingly slender emergence (arrowhead) in the axil of a larger emergence. Scale bar = 1 mm.

The *Psilophyton* axes form dense mats of highly intertwined axes (Fig. 3F, G) resembling thickets of brambles. The plant material in these mats is robust: long sections of plant axes remained intact, except where disrupted by fractures in the host rock. Some of the longer axes that broke during processing still remained long enough to provide striking examples of the plant’s morphology (Fig. 4A, B) and robust enough that once dried they could be picked up and turned around between two fingers for examination (Fig. 4C, D).

The mats and axes therein, after being air-dried, present patterns of colour and are often flecked with siliceous debris that allow us to interpret their original orientation. Under reflected light the air-dried axes have an overall dark colour (mostly black) typical of carbonaceous material. Superimposed on this, upward-facing surfaces of the axes (upper side, hereafter) often bear light-coloured siliceous remains, especially in samples where the maceration was not taken to completion (e.g. Fig. 4C). Conversely, most downward-facing surfaces of the axes (lower side, hereafter) tend to have a matte black–brownish to amber coloration when dried (e.g. Fig. 4D, E) and rarely exhibit the whitish siliceous precipitate. Axes positioned deeper in the mats often have approximately the same matte dark coloration on both upper and lower sides, but the lower sides always have more of the brownish to amber coloration.

### Emergences on the *Psilophyton crenulatum* axes

#### Emergence morphology

**Fig. 5.**
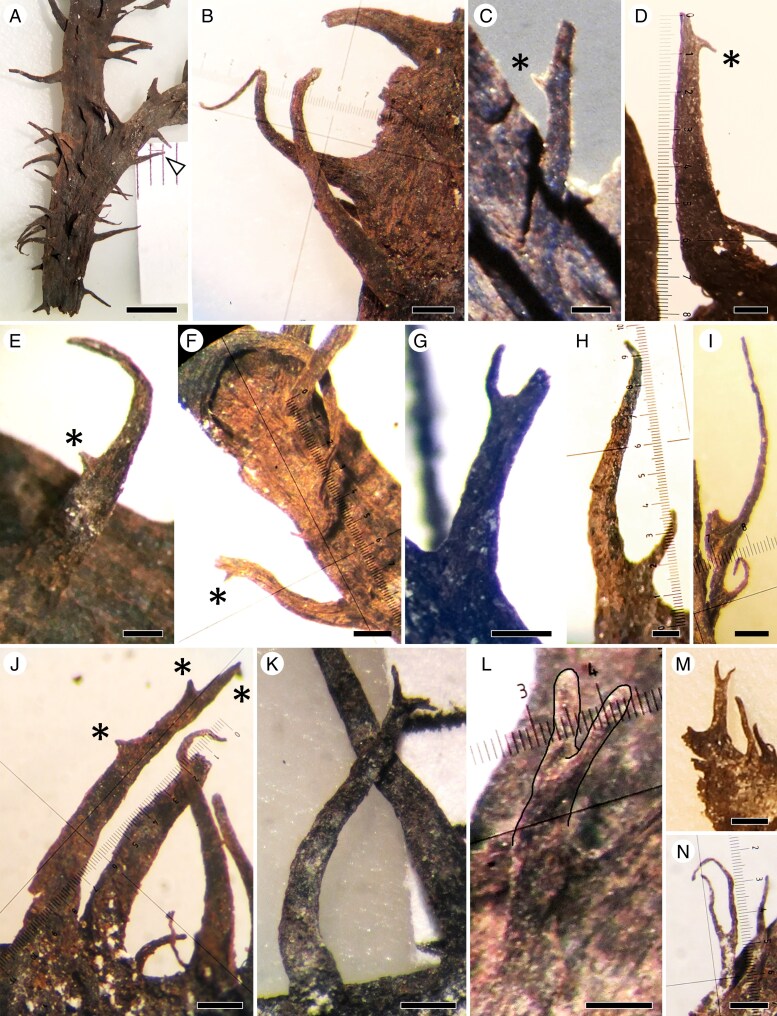
*Psilophyton crenulatum* simple and complex spinescent emergences. (A) Axis segment with stouter and broader-based simple emergences oriented in all directions; emergence at arrowhead detailed in Fig. 6F. Scale bar = 4 mm. (B) Simple emergence with helically coiled tendril-like tip ∼33–66 µm in diameter. Scale bar = 500 µm. (C–F) Emergences with small lateral outgrowths (asterisks). Note sinistral twisting of emergence with semicircular curvature and sinuous shape the emergence bearing the outgrowth, in (F). Scale bars = 300 µm. (G–I) Emergences with more developed lateral outgrowths. Note curved outgrowth close to the base of the emergence in (I). Scale bars = 300 µm. (J) Emergence with three lateral outgrowths (asterisks). Scale bar = 500 µm. (K–M) Emergences with apical dichotomy; outline of emergence in (L) traced for clarity. Scale bars = 500 µm (K and M), 300 µm (L). (N) Bifurcated emergence with long fine tips. Scale bar = 400 µm.

**Fig. 6.**
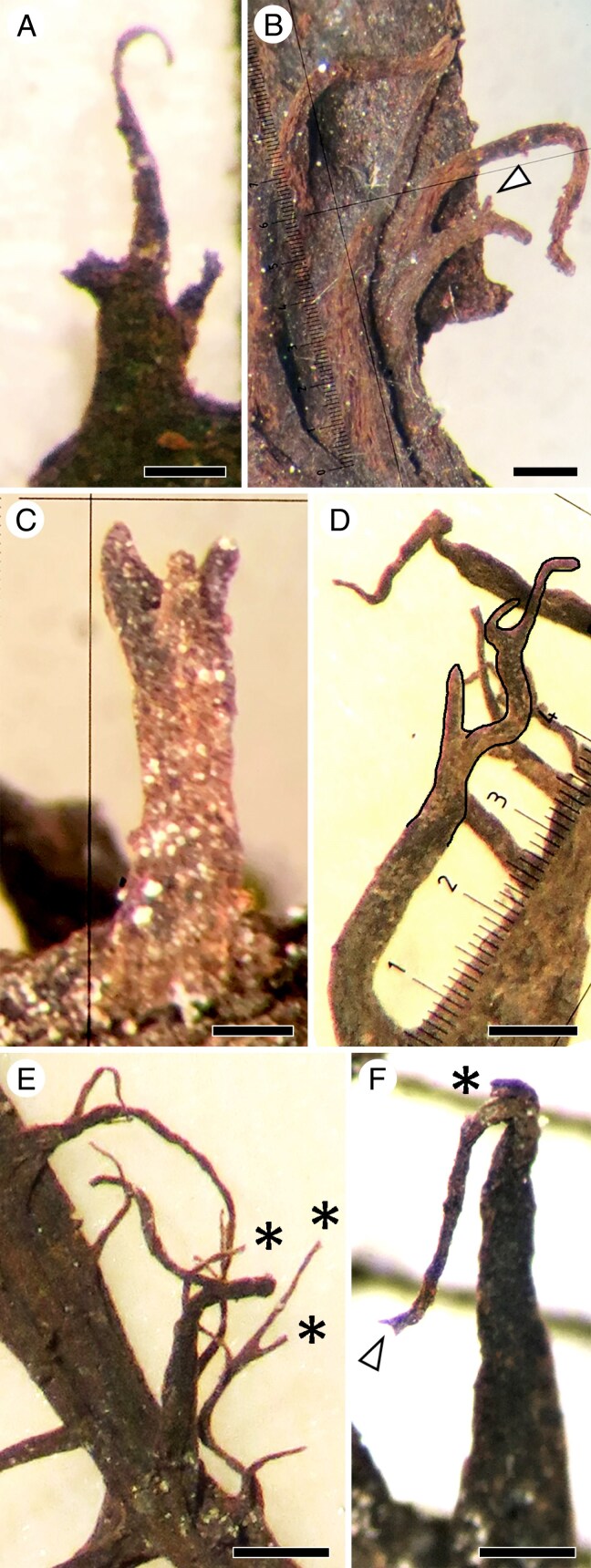
*Psilophyton crenulatum* complex spinescent emergences. (A) Bifurcated emergence with long fine tip (left tip broken); apparent right-pointing branch is a separate emergence in the background. Scale bar = 250 µm. (B) Emergence with two dichotomies; second dichotomy on the shorter branch at right has the left tip broken (arrowhead). Scale bar = 500 µm. (C) Emergence with three developing tips. Scale bar = 300 µm. (D). Emergence with two successive dichotomies; outline partially traced for clarity. Scale bar = 500 µm. (E) Delicate sinuous emergence with two dichotomies producing branches with long fine tips (asterisks); central tip has an incipient dichotomy. The apparent right-pointing branch at the base is a separate emergence in the foreground. Scale bar = 1 mm. (F) Detail of Fig. 5A. Emergence with a dichotomy producing two long fine tips; one is broken (asterisk) and the other has an incipient apical dichotomy (arrowhead). Scale bar = 500 µm.

The axes of *P. crenulatum* bear two types of spinescent emergences: simple (unbranched) emergences (Fig. 4E–J) and complex (branched) emergences (Figs 5C–N and 6A–F). Of the 2090 emergences recorded on the 28 randomly selected axes (Supplementary Data Tables S1 and S2), 719 (34.4 %) were complete. Among the complete emergences, 677 (94.2 %) were simple and 42 (5.8 %) were complex. An additional 20 incomplete emergences were recognized as complex because they exhibited at least one branching point. This brings the total to 62 identifiable complex emergences, which is an underestimate, since complex emergences broken below their lowest point of branching would not have been recognized as such.

Most emergences are compressed, but occasionally their bases, tips and central portions retain some three-dimensionality, adding critical details on emergence morphology and anatomy. When preservation permitted, we observed crenulations on some emergence bases. The crenulations did not extend further than ∼1.5 mm from the emergence base.

**Fig. 7.**
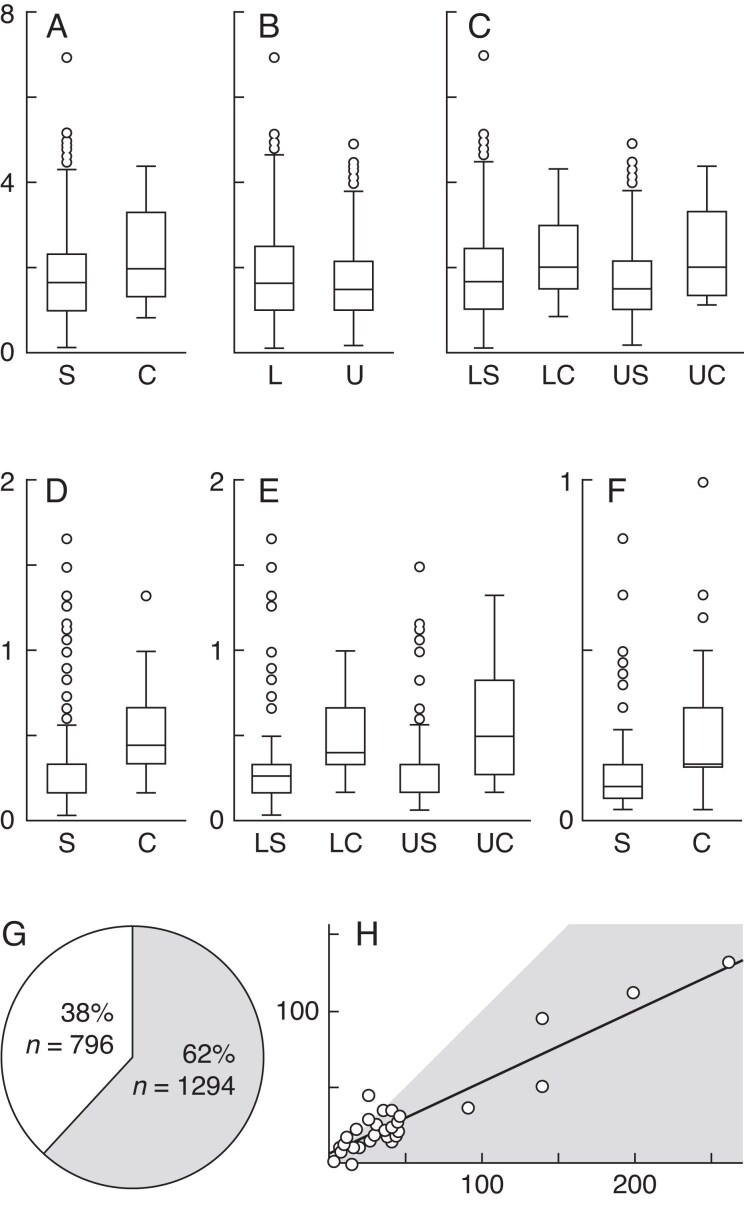
*Psilophyton crenulatum* emergence dimensions and distribution. (A–C) Length (on *Y*-axis, in mm) of simple (S) and complex (C) complete emergences on upper (U) and lower (L) sides of axes. (A) Length of total simple (*n* = 677) and all complex (*n* = 42) complete emergences on both axis sides. (B) Length of total complete emergences (simple and complex) on lower axis sides (L; *n* = 462) and upper axis sides (U; *n* = 257) complete emergences on both axis sides. (C) Length of complete emergences separated by lower versus upper axis side and by simple versus complex. (D) Size (on *Y*-axis, in mm) of complete emergences at the base, measured vertically along axes for total simple (S) and complex (C) emergences on both axis sides. (E) Size (on *Y*-axis, in mm) of complete emergences at the base, measured vertically along axes, separated by lower versus upper axis side and by simple versus complex. (F) Thickness (on *Y*-axis, in mm) of complete emergences at 1 mm from their base for simple (*n* = 673) and all complex (*n* = 42) emergences on both axis sides. Whiskers in (A–F) mark ±1.5 interquartile ranges; horizontal lines mark the medians; circles represent outliers. (G) Distribution of emergences between upper axis sides (white sector) and lower axis sides (grey sector). (H) Regression (*r*^2^ = 0.86) of numbers of emergences on upper (*Y*-axis) versus lower (*X*-axis) axis sides for 28 axes with a total of 2090 emergences; shaded area represents the domain of axes with fewer emergences on the upper than on the lower sides; only six of the 28 axes have a higher number of emergences on their upper sides than on the lower sides.

Emergence lengths (measured from base to farthest tip, for complex emergences) range between 0.1 and 6.9 mm (*n* = 719 complete emergences); of the incomplete emergences, one surpasses in length the longest complete emergence, measuring 10 mm. Compared with complex emergences, the simple emergences cover a broader length range (∼0.1–6.9 mm), albeit due to a few outliers, and include the longest emergences, but are significantly shorter on average (mean = 1.74 mm; *n* = 677) (Fig. 7A). Complex emergences range between ∼0.8 and 4.4 mm in length (mean = 2.31 mm; *n* = 42). There are no significant differences in emergence lengths on the upper sides (mean emergence length = 1.70 mm; *n* = 257) and lower sides of axes (mean emergence length = 1.81 mm; *n* = 462) (Fig. 7B). Consistent with this pattern, when compared separately within the upper and the lower sides of axes, complex emergences are longer than simple emergences, on average (Fig. 7C), but the differences are not significant. Long and short emergences are present on axes of all widths, so there seems to be no correlation between emergence length and axis size.

The emergences have circular to slightly vertically ovate bases of variable sizes that range between 0.03 and 1.65 mm. Many emergences have relatively broad bases, 1–2 mm in size, some of which approach the diameter of incipient axis branches (Figs 4C and 6B); other emergences are hair-like in their entirety or towards their tips (Figs 3D and 4G). Simple emergences are significantly thinner at their bases (mean base thickness = 0.30 mm; *n* = 677) than complex emergences (mean base thickness = 0.51 mm; *n* = 42) on both lower and upper sides of the axes (Fig. 7D, E). There is no significant difference in the sizes of emergence bases between the upper and lower sides of axes. Wider axes tend to have stouter emergences than the thinner axes, except on lower sides, where stouter and narrower spines are mixed.

Emergences are round in cross-section above the base, as they taper towards the tips. At 1 mm from the base, emergences range between 0.03 and 0.99 mm in thickness (mean thickness = 0.14 mm; *n* = 715), with most of them thinner than 0.35 mm and only a few thicker outliers (the thickest being a complex emergence on the upper side of an axis: 0.99 mm thick at 1 mm from the base). The same significant difference seen in the size of the emergence bases is seen in the thickness of emergences at 1 mm above their bases; simple emergences are thinner (mean thickness = 0.14 mm; *n* = 673) than complex emergences (mean thickness = 0.27 mm; *n* = 42) (Fig. 7F), with no significant difference in the thickness of emergences at 1 mm from the base between the upper and lower sides of axes.

Of the 719 complete emergences measured, 533 (74.1 %) have some portion of their total length that is <330 µm in diameter, i.e. filiform. Of those 533 emergences, almost three quarters [390 emergences (73.2 %)] have a filiform apex >1 mm, 291 emergences (54.6 %) have a filiform apex >1.5 mm, 101 (18.9 %) have filiform apices >2.5 mm, and 47 emergences (8.8 %) reach filiform lengths >3 mm; 12 emergences have filiform tips >4 mm. The tips of emergences taper to one or two cells wide (Fig. 4G–J) and range between 17 and 165 µm in width. Emergence tip widths average between 34 and 41 µm for simple and complex emergences on both upper and lower sides of the axes, with simple emergences on both sides of axes having broader tip width ranges (due to a few outliers) than the complex emergences.

In terms of orientation, of 719 complete emergences, only 76 (10.6 %) are straight, while the majority (643, 89.4 %) are curved, with an overall irregularly sinuous appearance (e.g. Fig. 4G–J). Specifically, 471 (65.5 %) of the 719 emergences are oriented apically: 148 on the upper side of axes and 323 on the lower side. Another 230 emergences are oriented horizontally, roughly perpendicular to the subtending axes (102 on upper sides and 128 on lower sides). Very few emergences point basally: 7 on upper axis sides and 11 on lower sides. The filiform apical portions of simple and complex emergences have no preferred curvature: some bend towards the apex of the axis, others towards the base, while some bend laterally with respect to the subtending axis or back towards it. The cells making up the filiform portion of some of the emergences, beyond 1.5 mm from the base, form a sinistral (counterclockwise) helix, as viewed from the tip of the emergence (Figs 4E, F and 5F).

Most of the complex emergences (39 out of 62 observed) branch only once, on average at 1.3 mm from the base. Some branch twice (Fig. 6B, D, F) or three times (Fig. 6E). One of the thrice-branched emergences is also the longest of the complex emergences, with 4.36 mm total length. Complex emergences have very diverse morphologies. Some have simple bifurcations, which can be located close to the base or more distally (Figs 5K–N and 6F); these may reflect relative developmental age of the emergences at the time of burial. The bifurcations can be relatively isotomous (Fig. 5K–N) but in most cases they are anisotomous (Figs 5C–J and 6D, E). Some of the emergences show incipient branching at the tips, either bifurcations (Fig. 6F) or trifurcations (Fig. 6C). The most dramatic of the complex emergences are those that resemble small root-like structures displaying multiple points of asymmetric division (e.g. Fig. 6D, E).

The complex emergences are branched in highly irregular patterns and, when preserved more completely, their branches have long-tapering filiform terminal portions. Some of the complex emergences could be taken for simple emergences but for the single or multiple small lateral outgrowths that they bear (Fig. 5C–J). The lateral outgrowths can be located close to the base of the emergences or randomly dispersed along their length. Some of the outgrowths seem to be pubescent (Fig. 5C, J), while others range from subtle bumps to longer outgrowths with filiform tips (Fig. 5D–I), possibly illustrating developmental stages. Indeed, some anisotomous bifurcations likely represent the divergence points of more developed lateral outgrowths.

#### Emergence anatomy

**Fig. 8.**
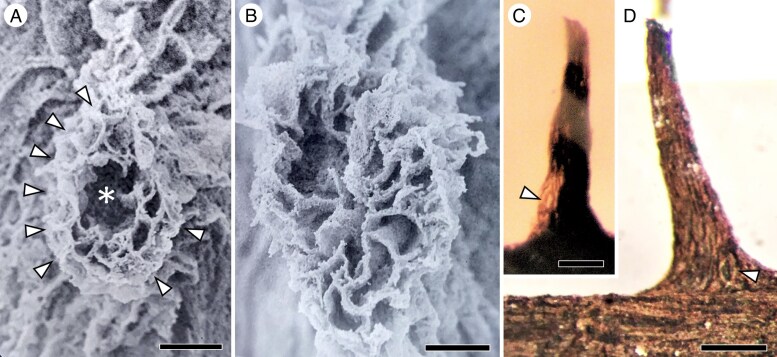
*Psilophyton crenulatum* emergence anatomy. (A, B) Bases of broken emergences showing internal anatomy. Arrowheads in (A) indicate anticlinal walls of torn epidermal cells. Note overall thin walls of the emergence cells and central void [especially conspicuous in (A); asterisk] probably left by decomposition of more delicate parenchyma. Scale bars = 50 µm (A), 30 µm (B). (C, D) Ovoid cell patterning in epidermis at base of emergences (arrowheads) possibly corresponding to stomata or substomatal chambers. Note amber-coloured anticlinal walls of torn epidermal cells in (D). Scale bars = 200 µm (C), 400 µm (D).

Cells on the surface of emergences are continuous with those on the surface of axes; they have the same shapes, sizes and orientations (e.g. Figs 4E and 8A). It is possible that an epidermal layer consisting of thin-walled cells that was present on the emergences and axes was preserved only occasionally. This is suggested by two observations. One is that some areas on emergences and axes, especially on lower sides, are covered by thin patches of lighter-coloured, brownish to amber material (e.g. Figs 4D, E, 5B and 8D) that could represent remnants of the epidermis or cuticle. The second observation comes from emergences that are broken, revealing the internal anatomy (Fig. 8A, B). In many cases, the surface of those emergences is covered in regular ridges that protrude radially from the surface of the emergence (Fig. 8A, B). The distances between the ridges are comparable to the sizes of neighbouring cells, indicating that the ridges are anticlinal walls of epidermal cells that lost their outer periclinal walls. Patches of periclinal walls (with attached anticlinal wall fragments) detached from the epidermis would correspond to the patches of amber-coloured material present in some areas on axes and emergences (Figs 4E and 8D). Small oval dimples on the surface (Fig. 8C, D) may correspond to stomata or substomatal chambers, but preservation precludes validating this interpretation; such features were not observed beyond ∼1.5 mm from the base of emergences.

Irrespective of the presence of an epidermal layer of thin-walled cells, the outermost preserved cells of the emergences have relatively thin walls, 1–5 µm thick (Fig. 8A, B). The anatomy of the emergences consists of two or three layers of such cells, which are 8.5–20.5 µm in size (average 13.2 µm) in cross-section. The centre of the emergences has a void which may have been occupied by thin-walled parenchyma cells (like the case documented in *Psilophyton diakanthon*; Colston *et al*., 2023), which were not preserved.

Our material preserves some evidence consistent with presence of both diffuse (elongation) and apical growth in the *P. crenulatum* emergences. Diffuse growth is suggested by the intermixing of smaller rounded to oval cells (30–150 µm in length) with much elongated cells (≥360 µm) that are tapered sharply at both ends (Fig. 4F). This bimodal cell shape and size pattern is especially conspicuous toward the tips of emergences, where they taper to few cells in thickness. Conversely, some complex emergences likely represent stages of apical branching and subsequent growth, which would imply at least some contribution from apical meristematic growth. Such are appendages with multiple ‘bumps’ at the tips (Fig. 5D, J), which could represent early stages of apical branching, and others that might represent later stages (Fig. 5E, G–I) as they bear short, small branches at the tips. Subsequent development from such apical branching points would lead to the morphologies seen in more developed complex emergences (e.g. Fig. 6B–E). All this evidence suggests a developmental scenario for the *P. crenulatum* emergences that included apical regions with meristematic functions (Figs 5K and 6F), responsible for apical branching and some elongation, and further increase by diffuse growth that continued for some period of time behind the apices. This pattern implies prolonged, albeit probably not strictly indeterminate, growth that resulted in complex irregular morphologies.

#### Emergence distribution on axes

The distribution of *P. crenulatum*emergences on axes is vertically polarized: emergences are significantly more numerous on the lower sides of axes than on the upper sides. Of the 2090 total emergences recorded on 28 axes, almost two thirds (1294; 62 %) are found on lower axis sides and 796 (38 %) on the upper sides (Fig. 7G; Supplementary Data Table S2). Additionally, the roughly 2:1 ratio between numbers of emergences on lower versus upper axis sides is a strong correlation among all axes analysed: *r*^2^ = 0.86. When considered individually, only 7 of the 28 axes have equal or higher numbers of emergences on their upper sides than on the lower sides (Fig. 7H). Of the other 21 axes, one has spines only on the lower side (axis 6), and the other 20 axes have on average 1.8 times as many emergences on their lower sides than on the upper sides (see also regression line in Fig. 7H). The subset of 719 complete emergences shows the same pattern of distribution: 257 of these emergences (36 %) occur on upper sides of axes and 462 emergences (64 %) occur on lower sides.

Emergence distribution by type (simple versus complex) shows no vertical polarization. Both upper and lower sides of axes have the same 16:1 ratio of simple to complex emergences: 242 simple and 15 complex emergences on upper sides, and 435 simple and 27 complex emergences on lower sides.

**Fig. 9.**
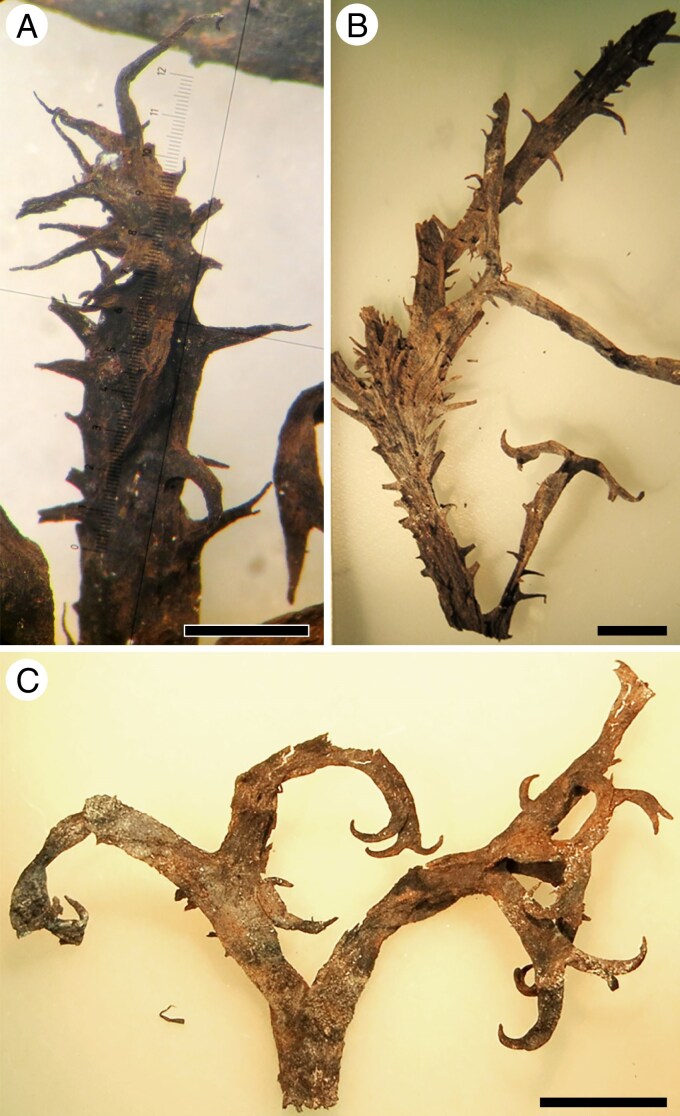
*Psilophyton crenulatum* emergence distribution. (A) Detail of Fig. 4A. Developing axis tip bearing 31 emergences in the terminal segment and mostly naked below it. Scale bar = 1 mm. (B) Axis with 46 emergences coming off the lower surface in an area of profuse branching; only 10 emergences are present on the upper surface. Scale bar = 5 mm. (C) Upper side of vegetative truss with closely spaced apical dichotomies and mostly devoid of emergences. Scale bar = 5 mm.

A few axes exemplify the patterns of longitudinal distribution of emergences. Axis 4 (Fig. 9B, Supplementary Data Table S2) bears 46 emergences on the lower side over a 15-mm segment basal to and including a zone of profuse branching. Only five of these emergences are found along the basal 5 mm of the segment, where the axis is 3 mm wide, with the remaining 41 emergences on the lower surface occupying the apical 10 mm of the segment, where the axis reaches 5 mm in width. In contrast to the lower side, the upper side of axis 4 only bears ten emergences along the same 15 mm segment. Axis 4 highlights the distributional disparity in zones of profuse branching where, in this instance, there are four times as many emergences on the lower side compared with the same region’s upper side. Another axis (axis 9; Figs 4A and 9A, Supplementary Data Table S2) has several growing tips, of which one, only 1.5 mm wide, has 31 emergences on its lower side within 7 mm from the apex.

Vegetative and fertile lateral trusses bear fewer emergences than the major axes subtending them. For example, axis 30 (Fig. 9C, Supplementary Data Table S2), a vegetative truss attached to a larger axis, has five or six dichotomies that terminate in recurved tips. This truss is ∼20 mm in length and spans ∼30 mm in width, but there were only five emergences on the entire truss. In general, lateral trusses, both vegetative and fertile, have a paucity of emergences when compared with major axes.

Overall, the highest density of emergences occurred (1) on lower axis sides, (2) on lower sides in areas of profuse branching of axes, and (3) in the vicinity of growing tips. All these distribution patterns were independent of axis width.

## DISCUSSION

### Stratigraphy, lithology and taphonomy support *in situ* preservation of *Psilophyton crenulatum* mats

Doran (1980) originally described the habitat of *P. crenulatum* as a lacustrine or back-levee floodplain environment where the plants formed dense populations. Subsequent publications on the geology and depositional environments of the Richardsville Member of the Val d’Amour Formation provided additional detail on the depositional environments of the layers that preserve *P. crenulatum*. The coaly shales in the Thompson Brook Member, which underlies the Richardsville Member, are interpreted as terrestrial wetland deposits (based on absence of marine palynomorphs; Wilson *et al*., 2004), where deposition in small lakes and marshes was interrupted by major flood events that laid down gravel and sand (Kennedy *et al*., 2013). Gensel *et al*. (1997, 2005) describe the recessive sequence of the Richardsville Member just above the *P. crenulatum* lenses as volcaniclastic sedimentary rocks that are bounded by two distinct rhyolite flows (Fig. 1C). Plant fossils are abundant in this recessive sequence, suggesting that the hiatus between eruptions was long enough for the area to be revegetated after emplacement of the subjacent rhyolite flow (which contains the *P. crenulatum* lenses; Fig. 1C). These reports provide evidence that the local terrain preceding and succeeding the fossilization of *P. crenulatum* was, in general, a wetland. It is therefore likely that these wetlands also existed during the time of *P. crenulatum*.

The lenses containing *P. crenulatum* are bounded by rhyolite and petrographic analysis identified these plant-bearing layers as a secondarily silicified tuff. The lack of discernable erosion immediately above or below the *P. crenulatum* lenses (Fig. 2A) suggests that the plant assemblage was not transported and represents populations preserved *in situ*. The secondary silicification could be the result of hydrothermal activity, which is thought to have been present in and around the environments represented in the Richardsville Member (Wilson *et al*., 2004). Wetlands of equivalent age, where hydrothermal activity influenced the depositional environments and preservational outcomes, are represented in the Rhynie and Windyfield cherts in Scotland (Rice *et al*., 2002), which lie along the same major subduction zone as the *P*. *crenulatum* locality (Fig. 1A).

Aside from the above considerations, *in situ* preservation of the *P*. *crenulatum* material is supported by several aspects of the plant assemblage that are inconsistent with transport prior to burial. First, the plant remains form thick, dense mats of highly intertwined axes (Fig. 3F, G) devoid of other plant taxa. This is inconsistent with transport, which typically mixes plant fragments from different sources and multiple taxa. Second, the *P*. *crenulatum* axes are extensive and mostly intact with little or no disarticulation, such as that engendered by transport. Third, the plant material preserves delicate features (meristematic areas and fine hair-like apical portions of emergences) that would have been obliterated during transport.

It could be argued that *in situ* preservation is inconsistent with the absence of (1) plant axes cross-cutting the bedding and (2) palaeosol layers underneath the *P. crenulatum* mats. (1) If *P. crenulatum* lacked rooting structures that penetrated the substrate to any significant extent, we would not expect evidence of axes cross-cutting the bedding downwards; if the upright parts (e.g. sporangium-bearing axes preserved in the mat) bowed under the weight of the volcanic ash that buried the plants, we also would not expect upright axes cross-cutting the bedding. (2) Palaeosol layers are not always present underneath plant communities preserved *in situ*, especially if the plants did not have deep-penetrating rooting structures or if the communities were not long-lived in a given location. For example, in the latest Lochkovian–earliest Pragian Beartooth Butte Formation, populations of the lycopsid *Sengelia* unequivocally preserved *in situ* (Matsunaga and Tomescu, 2017) have no palaeosol underneath. In summary, based on these considerations and the evidence discussed above, *in situ* preservation is the best supported interpretation of the taphonomy of the *P. crenulatum* mats.

If the *P*. *crenulatum* assemblage is preserved *in situ*, then its stratigraphic and sedimentary context informs the plants’ living environments. Petrographic thin sections do not show any discernible pedogenic or boundary layer between the lens containing the plant material and the underlying rhyolite. This suggests that the plants were growing either on a thin covering of ash or in a shallow but wet and slightly sandy depression in the pre-existing rhyolite bed. Because the Wilson *et al*. (2005) report describes nearby hydrothermal activity in the Richardsville Member hosting the *P*. *crenulatum* assemblage, it is possible that the plants populated a hydrothermal wetland, where they were covered by ashfall. The ashfall likely consisted of cooled volcanic ejecta, rather than originating from superheated pyroclastic clouds, an interpretation supported by the preservation of fine and delicate structures on the *P. crenulatum* axes; if the ashfall had somewhat higher temperature, that could have been attenuated sharply by the density and thickness of the plant mats. The paucity of intact epidermal cells on the *Psilophyton* axes suggests that the hydrothermal activity had temperatures and pH levels that could strip away the delicate epidermal layers (Channing and Edwards, 2004).

### The emergences of *Psilophyton crenulatum* probably functioned as rooting structures

The hypothesis that the emergences of *Psilophyton crenulatum* had a rooting function is supported by evidence coming from their morphology and distribution *on axes* and is consistent with the absence of any other types of rooting structures associated with the *P. crenulatum* mats. These considerations do not necessarily exclude functions other than rooting, for the emergences, especially on aerial portions of the plant. The focus here, however, is on rhizomatous axes, where we found the greatest disparity in emergence distribution between upper and lower sides.

The most convincing evidence for rooting function is the strongly polarized vertical distribution of emergences: the lower sides of axes bear on average almost twice as many emergences (and up to four times as many emergences in areas of profuse branching) compared with the upper sides (Fig. 7G, H). The strong correlation of emergence distribution between lower and upper axis sides across multiple axes showing a broad range of emergence densities, even when the few axes that show inverse polarization of emergence distribution are included (Fig. 7H), indicates that the polarized distribution is a consistent pattern and not a random occurrence. In turn, this suggests that the majority of axes sampled represent rhizomatous portions of *P. crenulatum* and that the few axes with similar or higher numbers of emergences on upper than lower sides may represent upright axes of the plant preserved in the mats that are dominated by rhizomatous axes.

The vertical polarization of emergence distribution, which also supports *in situ* preservation of the *P. crenulatum* axes, is consistent with the attachment and absorptive functions we expect for rooting structures. Alternative explanations of the polarized distribution of emergences on axes are less parsimonious. For instance, a role of the emergences in connecting non-self-supporting upright neighbouring axes to each other would produce a more even distribution around the axes. Potential roles in increasing photosynthetic surface area, maintenance of an air layer buffering temperature variations or water loss, or shading the axes against excessive light would require higher emergence densities on the upper sides of rhizomatous axes than on lower sides, or even distribution around upright axes. Furthermore, comparisons with trichomes are not warranted because the emergences of *P. crenulatum* are complex extensions of the axis tissues, as demonstrated by their anatomy, and not just simple epidermal appendages, like the trichomes.

In the context of sporophytes that had significant portions with rhizomatous growth it is also worth noting that areas of profuse branching along the axes had consistently higher density of emergences (Figs 4H and 9A, B). This distribution pattern suggests that *P. crenulatum* may have had stoloniferous growth habit, with rhizomatous portions exploring the substrate in search of areas with concentrated resources (water, nutrients), where the axes branched profusely generating tufted portions and produced more abundant rooting structures, which are especially numerous on the lower sides of axes in these positions.

Indirectly supporting a rooting role for the emergences of *P. crenulatum* is the absence from the fossiliferous layers of any other types of rooting structures that could have been associated with the plants. The underlying layers do not preserve any evidence of axes with positive gravitropism penetrating downwards from the plant mats. Within the mats, the careful disassembly process demonstrated an absence of axes running downwards, perpendicular to the general horizontal orientation of the axes, and absence of K-branching. Additionally, the presence of numerous emergence tips 22–130 µm in diameter (e.g. Figs 4G, I, J and 6A), similar in size to rhizoids of coeval plants (Table 1), demonstrates that the *P. crenulatum* material has good potential for preserving such structures. Yet we found no typical rhizoids along the axes, despite careful processing and observation.

The morphology of *P. crenulatum* emergences provides additional characters that are consistent with a rooting function rather than with any alternative interpretation. Many of them, both simple and complex types, are irregular in shape and sinuous (e.g. Fig. 4G, I, J), sometimes with helically coiled tips (e.g. Fig. 5B). This, along with the irregular branching of all the complex emergences observed, fits the morphology of organs that grow through the heterogeneous material that is the substrate of plants, with irregularly distributed clasts, mineral grains and porosity.

While not providing direct evidence for a rooting function, some developmental aspects of the *P. crenulatum* emergences are consistent with it. Such are their potential for apical meristematic growth and branching (e.g. Figs 5K–N and 6F) and their capacity to branch from dormant or delayed meristems. The latter is suggested by the presence on some emergences of small lateral ‘bumps’ interpreted as branch primordia (e.g. Fig. 5C, J) that contributed to their irregular branching pattern. However, the meristematic growth of the emergences seems to have been limited, because they terminate in long, fine rhizoid-like and rhizoid-sized tips (e.g. Figs 4G, I, J and 5B, N), which suggest an absorptive function, consistent with a role in rooting.

Finally, the anatomy of emergences does not reject an absorptive function. The cells in their outer layers are thin-walled (Fig. 8A, B), consistent with an absorptive role and in contrast to the cells that form outer layers of emergences with demonstrated protective function (e.g. *Psilophyton diakanthon*; Colston *et al*., 2023). While *P. crenulatum* emergences lack conducting tissues, their hypothesized core of parenchymatous cells could have transferred to the subtending axes water and nutrients absorbed through the rhizoid-sized tips, similar to the connecting tissue described by Kerp *et al*. (2001) in *Nothia aphylla*, which was compared to a transfusion-type tissue by Kenrick and Strullu-Derrien (2014).

The combined weight of all the evidence discussed above supports the hypothesis that the emergences of *P. crenulatum* had a rooting function. Additionally, since to our knowledge similar analyses have not been performed on any other early tracheophytes, the finding of this high degree of variation in emergence morphology and this type of distribution is novel in itself and the conclusions that can be drawn from this analysis are unique, regardless of the functional interpretation.

### Evolution of rooting structures

#### What do we know about early rooting structures?

We classify the broad diversity of early polysporangiophyte and tracheophyte rooting structures into two main morphological types. One type comprises non-vascularized multicellular structures that project outwards from the plant axes forming ridges, domes, etc. and are densely covered in rhizoids. These specialized structures, hereafter referred to as rhizoid-bearing projections, are part of what Kenrick and Strullu-Derrien (2014) have referred to as rhizoid-based rooting systems. The second type consists of branches of different developmental origin and homology (Table 1).

Rhizoid-bearing projections, produced typically on rhizomatous portions of plant axes, are known in Pragian protracheophytes and rhyniopsids: *Aglaophyton majus* (Edwards, 1986; Remy and Hass, 1996; Hetherington and Dolan, 2017*a*), *Rhynia gwynne-vaughanii* (Kidston and Lang, 1917; Hetherington and Dolan, 2017*a*) and *Nothia aphylla* (Kerp *et al*., 2001; Kerp, 2017). In *Horneophyton lignieri* (Kidston and Lang, 1920*b*; Hetherington and Dolan, 2017*a*) the rhizoids are borne on inflated, lobed plant bases. The rhizoids in all these plants are unicellular and range from 20 to 50 µm in diameter.

Branches with rooting function are borne on undifferentiated axes or on stems. We grouped them in three categories: (1) rooting axes produced by K-branching; (2) branches developed from subaxillary tubercles located below branching points of axes; and (3) branches whose divergence is not associated with branching points of subtending axes or stems. Whereas the first and third type are known in both zosterophylls and lycopsids [and the last type has also been reported in one rhyniopsid, *Stockmansella* (*Taeniocrada*) *langii*; Schweitzer, 1980, Fairon-Demaret, 1985, 1986], branches developed from subaxillary tubercles are limited to zosterophylls.

The oldest (Lochkovian) and longest-ranging (through the Eifelian) branches with rooting function are the rooting axes produced by K-branching (also known as H-branching). These are often positively gravitropic and typically produced on rhizomatous portions of the plants. Among zosterophylls rooting axes produced by K-branching are known in *Zosterophyllum myretonianum* (Walton, 1964), *Z. shengfengens*e (Hao *et al*., 2010), *Tarella trowenii* (Edwards and Kenrick, 1986), *Bathurstia denticulata* (Kotyk and Basinger, 2000; Gensel *et al*., 2001), *Sawdonia ornata* (Rayner, 1983) and possibly additional species (Gensel *et al*., 2001); they have also been documented in the putative zosterophyll *Hsua robusta* (Li, 1992) (Table 1). Among the lycopsids, rooting axes produced by K-branching are known in *Sengelia radicans* (Matsunaga and Tomescu, 2016, 2017), *S. devonica* (Schweitzer and Giesen, 1980; Gensel *et al*., 2001; Hao and Xue, 2013; Matsunaga and Tomescu, 2017) and *Drepanophycus spinaeformis* (Rayner, 1984). In some of these lycopsids the rooting axes bear reduced leaves indicative of stem homology.

Branches with rooting function developed from subaxillary tubercles (or the subaxillary tubercles they originate from) are known in plants that span the late Lochkovian–early Pragian through the Emsian (Table 1): *Thrinkophyton formosum* (Kenrick and Edwards, 1988), *Deheubarthia splendens* (Edwards *et al*., 1989), *Gosslingia breconensis* (Edwards, 1970; Edwards *et al*., 1989), *Margophyton goldschmidtii* (Kenrick and Edwards, 1988), *Anisophyton gothani* and *A. potoniei* (Remy *et al*., 1986*a*, *b*) and *Crenaticaulis verruculosus* (Banks and Davis, 1969).

Branches with rooting function not associated with other branching points of the subtending axes or stems have been reported as early as the Pragian in the zosterophylls *Bathurstia denticulata* (Kotyk and Basinger, 2000; Gensel *et al*., 2001), *Crenaticaulis verruculosus* and possibly another zosterophyll species (Gensel *et al*., 2001), in *Hsua robusta* (Li, 1992) and in the lycopsids *Drepanophycus spinaeformis* (Gensel *et al*., 2001) and *Asteroxylon mackiei*. In the latter, such branches may bear reduced leaves (Kidston and Lang, 1920*a*, 1921; Hetherington *et al*., 2021), indicative of stem homology.

Structures interpreted as true roots (Table 1) have been reported in the Early Devonian only in the late Lochkovian–early Pragian lycopsid *Sengelia radicans*, in which they are non-gravitropic and arise from rooting axes produced by K-branching (Matsunaga and Tomescu, 2017), and in the Emsian *Drepanophycus qujingensis* (Li and Edwards, 1995), where they would correspond to shoot-borne roots. Positionally and morphologically comparable structures are present in the younger (Eifelian) *Sengelia devonica* (Schweitzer and Giesen, 1980; Gensel *et al*., 2001), whereas in the Pragian *Asteroxylon mackiei* the rooting branches with stem homology produced weakly gravitropic branches by anisotomous dichotomy (Hetherington *et al*., 2021).

The euphyllophytes are conspicuously absent in the deep fossil record of rooting structures (Table 1). The oldest euphyllophyte structures discussed as potentially having rooting function were described in the Emsian *Psilophyton dawsonii* by Banks *et al*. (1975) , who documented three slender lateral branching systems (‘branchlets’) in a region of profuse branching on a *P. dawsonii* axis. The increase in axis and xylem size in that region (Banks *et al*., 1975, Plate 24, Figs 62–65) prompted their speculation that the branchlets could represent ‘a first attempt to evolve a new function’ (Banks *et al*., 1975, p. 113) as precursors of root-like organs. Banks *et al.* based this hypothesis on comparison with a *Botryopteris antiqua* stem that showed noticeable increase in the amount of primary xylem and the number of protoxylem strands where it bore adventitious roots. However, the *Botryopteris* comparison is inconclusive because (1) areas of profuse branching are typically associated with increase in stele size and added complexity of protoxylem architecture irrespective of the identity of the organs attached (whether roots or otherwise); and (2) the *P. dawsonii* branchlets were borne on a sporangium-bearing aerial portion of the plant and not attached to an underground or rhizomatous axis. Thus, it is unfortunate that this unique reference to ‘roots’ in early euphyllophytes has persisted unchecked in the root evolution literature.

Aside from the *Psilophyton dawsonii* case, the oldest euphyllophyte rooting structures have been reported in Eifelian cladoxylopsids (Giesen and Berry, 2013): the appendages of *Calamophyton primaevum* interpreted as true roots lack anatomical preservation that would demonstrate anatomy typical of roots, such as actinostelic organization, exarch xylem maturation or endogenous origin from the subtending plant parts. Rooting appendages of a younger (Frasnian) cladoxylopsid positionally equivalent to those of *C. primaevum* and exhibiting better anatomical preservation also lack evidence for exarch xylem maturation and their root homology is unclear (Stein *et al*., 2023). The oldest unequivocal true roots have been described in the Frasnian cladoxylopsid *Xinicaulis lignescens* (Xu *et al*., 2017) and by Famennian time roots were also present in the cladoxylopsid *Pietzschia levis* (Soria *et al*., 2001; Soria and Meyer-Berthaud, 2004) and in several other euphyllophyte groups (Hetherington *et al*., 2020).

#### How does *Psilophyton crenulatum* fit among early tracheophyte rooting structures?

Structures with demonstrated rooting function are known among Early Devonian tracheophytes only outside the euphyllophytes (Table 1). The emergences of *P. crenulatum* interpreted here as rooting structures are different from the two morphological categories of rooting structures present in those coeval plants. Elongated-conical and spine-like in general aspect, they differ from the ‘lumpy’ rhizoid-bearing projections of the first category. Nevertheless, like those projections, they are not vascularized and have limited growth potential. Additionally, although there is no evidence that the emergences bore rhizoids, they terminate in rhizoid-sized tips. Compared with the axial rooting organs in the second morphological category, the *P. crenulatum* emergences are significantly smaller, relative to their subtending axes, and they lack vascularization. Nevertheless, like those axial rooting organs, they grew from an apical meristem that also allowed them to branch. Their geometry and relatively small sizes suggest that growth ceased by progressive depletion of the apical meristem and terminated in production of the rhizoid-sized tip.

Kenrick and Strullu-Derrien (2014) discussed the apparent differentiation in some Rhynie chert plants of parenchymatous tissue with putative transfer function in areas of the rhizoid-bearing projections underlying the rhizoids. Such tissues could have connected the projections to the vascular tissues of the axes. If the emergences of *P. crenulatum* had a rooting function, as proposed here, they could have functioned without vascular tissues, given their small size. Instead, parenchyma cells probably present at their centre could have had enhanced water transfer function similar to those reported in the Rhynie chert plants. In this case, the emergences of *P. crenulatum* represent a *sui generis* type of rooting structure that had no close analogue among Early Devonian tracheophytes.

Positive gravitropism in response to gravisensing is an important shared feature of many roots and rooting structures. The significantly higher frequency of emergences on the lower sides of *P. crenulatum* axes indicates a vertical polarization of the regulators responsible for emergence initiation, which, in turn, suggests a development response to the gravity signal (possibly in combination with response to contact with the substrate). Such a response could have been auxin-mediated, similar to the mechanism proposed for the production of rhizoid-bearing projections in protracheophytes and rhyniopsids (Jones and Dolan, 2012; Kenrick, 2002, 2013; Hetherington and Dolan, 2017*a*). If that was the case in *P. crenulatum*, then this would represent the oldest evidence for a gravity-induced growth response in the euphyllophyte clade.

#### Hypotheses on euphyllophyte root evolution

Aside from hypotheses proposed by Hetherington *et al*. (2020) on root branching, the tempo and mode of root evolution in the euphyllophyte clade have not been the subject of much discussion. If the emergences of *P. crenulatum* are rooting structures, they would be the oldest such structures known in the clade and could illustrate the earliest stage in euphyllophyte root evolution, which raises questions about the developmental changes involved in the evolution of roots from such structures.

Among the features shared by all modern plant roots, gravisensing was present in *P. crenulatum*, as suggested by the polarized distribution of emergences on its axes. The regulatory network for production of tip-growing filamentous cells with rooting functions (rhizoids, root hairs, caulonemata), conserved across land plants (Jones and Dolan, 2012; Tam *et al*., 2015), must have also been present in *P. crenulatum*. We speculate that the gravisensing responses leading to higher emergence density on the lower sides of axes could have also activated that regulatory network, leading to development of rhizoid-sized tips in the emergences. Apical growth, another root feature, was also present in the *P. crenulatum* emergences, albeit not indeterminate. Nevertheless, the emergences were capable of apical branching, foreshadowing the branching mode suggested for the oldest euphyllophyte roots (Hetherington *et al*., 2020).

Unlike roots, the emergences of *P. crenulatum* lack vascularization. Instead, they probably possessed central parenchyma, in which vascular tissue differentiation could have been driven by evolutionary changes in auxin fluxes related to apical growth. Elongation by apical growth is associated with polar auxin transport, which specifies vascular tissue precursors above certain concentrations (Sachs, 1969, 1981; Aloni, 2010). Given their small size and limited apical growth, auxin fluxes probably present in the *P. crenulatum* emergences would not have reached concentrations that elicit vascular tissue specification. Developmental changes leading to prolongation of apical growth and longer emergences would have generated associated polar auxin fluxes that could have eventually reached concentrations required for specification and differentiation of vascular tissue (as demonstrated in modern roots; Sabatini *et al*., 1999; Benkova *et al*., 2003; Friml, 2003; Augstein and Carlsbecker, 2018).

No mechanistic hypothesis exists explaining the evolution of endogenous root origin – the development of roots from the pericycle, endodermis or equivalent tissues of stems or other roots. It is hard to imagine developmental changes that would lead from exogenously produced structures, like the emergences of *P. crenulatum*, to structures that grow from endogenously specified meristems. In living plants, the regulation of root origination is well understood only in the allorhizic rooting systems of seed plants, in which roots are produced as branches on other roots. In contrast, all seed-free plants are homorhizic; except for the short-lived embryonic root, roots arise endogenously on stems (Tomescu and Matsunaga, 2019). This suggests that the first embryophytes with true roots were homorhizic, so constructing hypotheses on the evolution of endogenous root origin in euphyllophytes requires understanding the regulation of root production in living seed-free euphyllophytes. However, to date, no developmental study has addressed this topic. Thus, until we understand the regulation of root production in living seed-free euphyllophytes and new discoveries fill the Lochkovian–Eifelian gap in the fossil record of euphyllophyte rooting structures, connections between euphyllophyte true roots and the emergences of *P. crenulatum* will remain speculative and these emergences will remain an isolated case of *sui generis* early euphyllophyte rooting structure.

#### How do the rooting structures of *Psilophyton crenulatum* inform the big picture of root evolution?

Independent of speculation on developmental changes that could have led to evolution of roots from emergences like those of *P. crenulatum*, interpreting these emergences as rooting structures has implications for the big picture of rooting systems evolution. In terms of mode of evolution, the fossil record has confirmed the independent evolution of roots in euphyllophytes and lycophytes, and that true root-possessing members of the two clades rose from lineages that lack true roots (Kenrick and Crane, 1997; Friedman *et al*., 2004). In terms of evolutionary tempo, the roots (or potential roots) recognized to date in euphyllophytes are Eifelian in age or younger (Giesen and Berry, 2013; Xu *et al*., 2017; Stein *et al*., 2023), whereas in lycophytes roots are known as far back as the late Lochkovian–early Pragian (Matsunaga and Tomescu, 2017). In the broader context of rooting systems evolution, *P. crenulatum* brings the origin of rooting structures among euphyllophytes to a time interval roughly equivalent to the oldest rooting structures documented in lycophytes: the emergences of *P. crenulatum* are younger than the positively gravitropic axes of Lochkovian *Zosterophyllum* (Walton, 1964; Hao *et al*., 2010) (Table 1) by only 10–12 million years.

Different in morphology from rooting structures of all coeval plants, the emergences of *P. crenulatum* support independent origination of rooting structures in the euphyllophytes. We hypothesize that euphyllophyte rooting structures originated *de novo* as emergences produced exogenously on axes which, in response to the gravity signal, produced the emergences preferentially on their lower sides. Prolongation of apical meristematic activity of the emergences would have led to evolution of these structures into axial organs with apical branching, consistent with the ‘apical branching-first’ hypothesis of Hetherington and Dolan (2018) and Hetherington *et al*. (2020). The transition from relatively small emergences to larger axial organs and the associated amplification of auxin fluxes through these organs would have also promoted establishment of vascular tissues connecting them to those of subtending axes.

Actinostelic root organization with exarch xylem maturation is shared across the lycophyte and euphyllophyte clades. This is intriguing, given the broad diversity of stelar architectures of the axes that subtend them in the two clades and considering that roots evolved independently in the two clades, in early members that had contrasting anatomies. The root-less axes of the zosterophyll ancestors of root-bearing lycopsids had exarch xylem maturation, like the true roots that are thought to have evolved from them. However, the axes of the trimerophyte ancestors of the root-bearing euphyllophytes have centrarch xylem maturation, unlike roots, allowing for an explanation of euphyllophyte root origin by *de novo* evolution as a *sui generis* organ, from structures like the unvascularized emergence of *P. crenulatum*. At the same time, the homoplastic exarch actinostelic structure suggests that root evolution, although proceeding on independent paths, was directed by the same selective pressures and was canalized by the same developmental constraints across all tracheophytes, the hypotheses and evidence for which were discussed by e.g. Wardlaw (1928) and Boyce (2005). Adding to those discussions and given the importance of polar auxin transport in root development – at the intersection of meristem maintenance, gravitropic responses and root tissue patterning (including xylem maturation patterns) – we concur with Boyce (2005) that the shared acropetal pattern of overall polar auxin transport (proposed by Tomescu and Matsunaga, 2019) may be a key factor determining the exarch organization of the xylem in all roots. If that is the case, then similar developmental constraints, as well as selective pressures of a physiological nature (water transfer to the xylem, Wardlaw, 1928), are both responsible for the shared but independently derived exarch actinostelic anatomy of lycophyte and euphyllophyte roots.

## Conclusions

*Psilophyton crenulatum* is one of the best morphologically characterized early euphyllophytes. The morphology, development and distribution patterns of its emergences, documented in detail here, can be used to infer the potential functions of the emergences. Along with the absence of any other potential rooting structures in *P. crenulatum*, multiple features of the emergences are more consistent with a rooting function than with any other possible role. These features include (1) irregular morphology (including forms with complex branching, sinuous and sometimes helical shapes), consistent with growth within heterogeneous substrates; (2) external layers consisting of thin-walled cells that would allow for easy diffusion of water; (3) apical meristematic growth that allowed for prolonged (albeit limited) elongation and opportunistic branching; (4) termination in filiform rhizoid-sized tips; and (5) polarized distribution on the subtending axes, with significantly more emergences on the lower sides of axes compared with the upper sides.

The hypothesis that the emergences of *P. crenulatum* had a rooting function contributes to our knowledge of when and how rooting structures might have arisen in euphyllophytes, filling a gap in our knowledge of evolution in the lineage that gave rise to modern ferns, equisetopsids and seed plants. The surface emergence-based rooting system of *P. crenulatum* has no close analogue among early tracheophytes and is best interpreted as a *sui generis* type of rooting structure, derived *de novo* in the euphyllophyte clade. If true roots evolved from such structures, then this evolutionary scenario fits previous hypotheses of stepwise evolution and of lateral branching preceded by apical branching in euphyllophyte rooting structures (Hetherington and Dolan, 2018; Hetherington *et al*., 2020).

If the emergences of *P. crenulatum* had a rooting function, they are the oldest known euphyllophyte rooting structures and represent a novel type among Early Devonian plants. The *P. crenulatum* emergences also provide the oldest direct evidence for gravity-induced morphological features in euphyllophytes. If euphyllophyte roots evolved from emergences like those of *P. crenulatum*, then they probably inherited from them apical growth and branching, and the capacity to express gene networks responsible for production of tip-growing filamentous cells. Progressive increase in size of such emergences could have increased auxin fluxes, leading to specification of vascular connections to subtending axes. Such significant and consistent auxin fluxes interacting with auxin-mediated gene regulatory networks assembled in stepwise fashion are thought to have generated, over evolutionary time, the root phenotypic diversity present across vascular plant lineages (Poelmans *et al*., 2025). These hypotheses will need to be tested through additional research on how roots develop in modern derived lineages and how their development is regulated, and by finding new fossils that can help fill the gaps in our understanding of timeline versus complexity in the evolution of euphyllophyte rooting structures.

## Supplementary Material

mcaf121_Supplementary_Data

## SUPPLEMENTARY DATA

Supplementary data are available at *Annals of Botany* online and consist of the following. Table S1: emergence morphometrics and morphology (Excel file). Table S2: total numbers of emergences on upper and lower sides of axes.
