# Supplementary material for: On the origin of euphyllophyte roots: hypotheses from an Early Devonian Psilophyton
Source: Ann Bot. 2025 Jun 13;137(6):1689–708. doi: 10.1093/aob/mcaf121 (PMC13274986; doi:10.1093/aob/mcaf121)
Supplement: mcaf121_Supplementary_Data [file mcaf121_supplementary_data.zip › DoranTomescu2024_SupplementaryTable2.docx]

**Supplementary Table 2.** Total numbers of emergences on upper and lower sides of axes.

| Axis number | Lower side | Upper side | Lower : upper side emergences |
| --- | --- | --- | --- |
| 1 | 26 | 29 | 0.90 |
| 2 | 45 | 28 | 1.61 |
| 3 | 7 | 11 | 0.64 |
| 4 | 45 | 22 | 2.05 |
| 6 | 15 | 0 |  |
| 7 | 20 | 11 | 1.82 |
| 8 | 27 | 15 | 1.80 |
| 9 | 42 | 25 | 1.68 |
| 10 | 37 | 22 | 1.68 |
| 11 | 16 | 11 | 1.45 |
| 12 | 139 | 51 | 2.73 |
| 14 | 36 | 35 | 1.03 |
| 15 | 8 | 8 | 1.00 |
| 16 | 30 | 19 | 1.58 |
| 17 | 10 | 13 | 0.77 |
| 18 | 38 | 18 | 2.11 |
| 19 | 261 | 132 | 1.98 |
| 20 | 139 | 95 | 1.46 |
| 21 | 44 | 19 | 2.32 |
| 22 | 12 | 18 | 0.67 |
| 23 | 31 | 26 | 1.19 |
| 24 | 41 | 15 | 2.73 |
| 25 | 46 | 31 | 1.48 |
| 26 | 91 | 37 | 2.46 |
| 27 | 26 | 45 | 0.58 |
| 28 | 18 | 23 | 0.78 |
| 29 | 41 | 35 | 1.17 |
| 30 | 3 | 2 | 1.50 |
| Total emergences | 1294 | 796 |  |
